# Mutations Matter: Unravelling the Genetic Blueprint of Invasive Lobular Carcinoma for Progression Insights and Treatment Strategies

**DOI:** 10.3390/cancers16223826

**Published:** 2024-11-14

**Authors:** Athanasios Kontogiannis, Eleftheria Karaviti, Dimitra Karaviti, Sophocles Lanitis, Georgia Gomatou, Nikolaos K. Syrigos, Elias Kotteas

**Affiliations:** 1 Oncology Unit, 3rd Department of Medicine, “Sotiria” Hospital for Diseases of the Chest, National and Kapodistrian University of Athens, 115 27 Athens, Greece; smd1800071@uoa.gr (A.K.); smd1800057@uoa.gr (E.K.); smd1900058@uoa.gr (D.K.); georgiagom@med.uoa.gr (G.G.); nksyrigos@med.uoa.gr (N.K.S.); 2 2nd Department of Surgery, Korgiallenio Benakeio Athens General Hospital, 115 26 Athens, Greece; drslanitis@gmail.com

**Keywords:** ILC, treatment, classification, histological subtypes, chemotherapy, endocrine therapy, drug resistance

## Abstract

Invasive Lobular Carcinoma (ILC) presents a distinct subtype of breast cancer, representing 10–15% of cases, with unique clinical and molecular features. Characterized by a non-cohesive, single-file invasion pattern, ILC is typically estrogen receptor (ER)- and progesterone receptor (PR)-positive but human epidermal growth factor receptor 2 (HER2)-negative. Despite favorable prognostic features, its highly metastatic nature and predilection for atypical sites contribute to lower long-term survival compared to invasive breast carcinoma of no special type (NST). ILC’s genetic landscape includes mutations in various genes (*CDH1, BRCA2, ATM*, etc.) and signaling pathways that impact treatment responses, especially in endocrine treatment. Furthermore, the diverse ILC subtypes complicate its management. Current challenges in chemotherapy, along with the targeted therapies, are also discussed. The present article aims to comprehensively review the recent literature, focusing on the pathological and molecular aspects of ILC, including associated genetic mutations influencing disease progression and drug resistance.

## Simple Summary

Invasive Lobular Carcinoma (ILC) of the breast is the second most common type of breast cancer. However, it is characterized by diverse morphological and clinical features. Most importantly, some subtypes are more aggressive and their responses to treatment may vary. These features are due to distinct genetic mutations that influence the behavior of these tumors. Given the importance of this cancer subtype, this article aims to provide an overview of the genetic mutations responsible for metastasis, drug resistance and prognosis, while also highlighting gaps in the current literature. The latest advances in treatment are also a main point of discussion and one with the most prospects for new research.

## 1. Introduction

Invasive Lobular Carcinoma (ILC) of the breast is the second most common type of breast cancer (BC) after invasive carcinoma of no special type (NST), formerly known as invasive ductal carcinoma (IDC), and its frequency ranges between 10% and 15% of all breast cancer cases in women [1]. The phenotype of ILC is typically characterized as positive for estrogen receptor (ER) and progesterone receptor (PR) and negative for the human epidermal growth factor receptor 2 (HER2), although a minority of HER2+ and triple-negative ILCs have also been described. These subgroups have therapeutic significance, with anti-HER2 agents and immunotherapy being potential treatment options for HER2-amplified and triple-negative tumors, respectively [2,3,4,5].

The typical scenario for ILC tumors displays favorable prognostic features, for instance, ER-positivity and slow proliferation rates. However, long-term overall survival tends to be lower for ILC, compared to NST patients [6,7]. This is due largely to the highly metastatic nature of the disease, which forms a very distinct pattern. Compared to NST, ILC tumors present metastases to the skeleton, gastrointestinal tract, peritoneum and gynecological sites, such as the myometrium, endometrium, cervix, and ovaries, more frequently. Lobular tumors are additionally correlated with an increased frequency of orbital metastases, which are generally rare and reported only in 2–3% of metastatic cancer patients [8,9]. Furthermore, the multicentric or multifocal nature of the tumor, combined with an increased risk of distant recurrence even after 5–10 years, as well as the larger size and higher nodal stage of the tumor, further complicate therapeutic outcomes, even in the setting of localized disease [10,11].

The diverse behavior of these tumors is due to their array of genetic characteristics (mainly gene mutations), leading to the heterogenicity of the disease. Typical for ILC are mutations in *CDH1*, the gene responsible for E-cadherin, resulting in loss of cell adhesion [7]. Besides *CDH1*, germline *BRCA2* mutations are additionally associated with a high risk of ILC development, whereas *ATM, CHEK2* and *PALB2* moderately predispose to ILC carcinogenesis [12]. Furthermore, a series of mutations predominantly found in ILC compared to NST tumors render ILC resistant to standard treatment, complicating therapeutic strategies. *HER2* mutations (HER2mut), APOBEC-derived mutations, FOXA1 and the WNT4 ligand contribute to resistance against endocrine treatment (ET) [13,14,15]. Similarly, FGFR1 has been correlated with tamoxifen resistance, and TP53 along with the PI3K/AKT/mTOR pathway have been associated with resistance to CDK4/6 inhibitors [16,17].

Therefore, the spectrum of the disease necessitates tailored treatment in both localized and metastatic settings. The purpose of this article is to comprehensively review the recent literature and assess the pathological and molecular background of ILC and the role of specific mutations in disease progression and drug resistance. Additionally, we discuss the recent advances in ILC treatment and its prospects, emphasizing the genetics of ILC and the potential differences between ILC and NST tumors regarding therapeutic outcomes.

## 2. Review Sections

### 2.1. General Clinicopathological Characteristics of ILC

Most frequently, ILC arises from pre-neoplastic lesions called atypical lobular hyperplasia (ALH) and lobular carcinoma in situ (LCIS), falling under the category of lobular neoplasia (LN). It is more commonly multifocal and bilateral than other primary breast cancers and is typically characterized by non-cohesive cancer cells invading the stroma in a single-file pattern, making it challenging to detect through physical examination or mammography [18]. Responsible for this feature are *CDH-1* gene mutations, located on chromosome 16q22 and encoding E-cadherin—a 120kDa single transmembrane glycoprotein that is considered pathognomonic for ILC and its subtypes. They result in loss of cell adhesion, often a result of biallelic inactivation of the gene [19]. The area of interest is presented with little desmoplastic response and the malignant cells appear similar to one another; they are small and round with little pleomorphism and uniformly distributed chromatin, without nuclei. The tumor cells may appear surrounding the duct or be signet ring-shaped. Target-like intracellular mucin accumulation is also possible [20].

Typically, ILC tumors present low grading—with 84% being classified as Grade 2—and lack vascular invasion [21]. They are often estrogen receptor (ER)- and progesterone receptor (PR)-positive, making them suitable for ET. However, some ILC cases do not follow this ER/PR+, HER2− pattern. Additionally, ILC tumors generally have lower tumor-infiltrating lymphocytes (TILs) compared to NST. High numbers of TILs (>10%) were present in approximately 15% of ILC cases, which was associated with poorer prognosis [22,23].

### 2.2. Histological Subtypes of ILC

**Figure 1:**
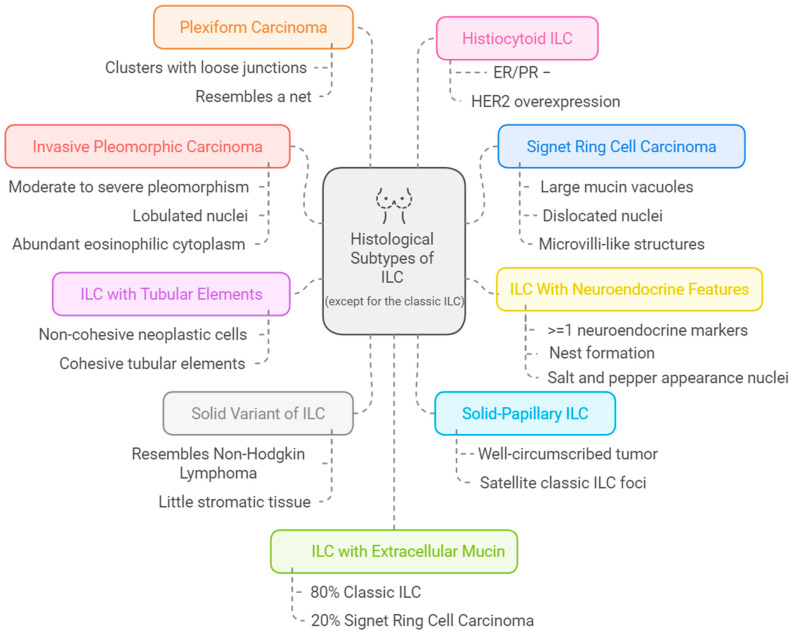
Overview of ILC histologic subtypes. Abbreviations: ILC: Invasive Lobular Carcinoma; IDC: Invasive Ductal Carcinoma.

According to WHO, less common subtypes have been described apart from the classic ILC subtype. Figure 1 provides a brief summary.

#### 2.2.1. Pleomorphic and Signet Ring Cell Carcinoma

Invasive pleomorphic lobular carcinoma (PLC) is a non-differentiated variant of ILC that is relatively rare (only 5% of ILCs) [24]. This subtype is divided into apocrine, histiocytic and signet ring cell types [25]. Tumor cells have sufficient pleomorphism (moderate to severe) and have medium-to-large-sized lobulated nuclei with well-distinguished nucleoli and abundant eosinophilic or granular cytoplasm. The area of interest may be necrotic and is often subjected to apocrine change. Signet ring cell carcinoma is also rare. The hallmark cells have large mucin vacuoles in their cytoplasm which dislocate the nuclei and give them a ring-shaped appearance. Microvilli-like structures plated to their interior can been seen under an electron microscope [19,24,26]. Apart from E-cadherin loss, PLC demonstrates losses in p53, BRCA1, BRCA2 and ESR, while showing high rates of *HER2* amplification. Laboratory findings suggest that inactivation of CDH1 and p53 may lead to PLC development [27]. On the other hand, 12.5–22.7% of PLCs are triple-negative. The aggressive nature of this ILC subtype may be due to negative prognostic factors (i.e., larger tumor size, lymph node involvement, increased metastatic potential and HER2 expression) rather than histology alone [27].

According to a population-based study by Haque et al., PLC histology is associated with reduced OS compared to IDC, as are factors like increasing age, poor grade and T and N stages. ER+ status was associated with improved OS, as was chemotherapy, radiation and endocrine treatment (ET) [28]. In the early-stage setting (T1-2 N0) there was no statistical difference in OS between ILC and PLC (5-year OS: 90.5% and 89.1%, respectively; *p* = 0.108). In advanced disease (T3-4 or N+), however, PLC patients showed decreased OS compared to IDC patients (5-year OS: 64.5% and 80.5%, respectively; *p* < 0.001) [28]. On the contrary, Narendra et al. found no statistically significant difference in PFS and OS between ILC and PLC. Factors associated with OS in PLC patients were surgical treatment, nodal status, tumor size and staging. Similarly, tumor size and nodal status were also associated with OS in ILC patients. These results are in line with the hypothesis that histology may not be independently associated with poorer prognosis in PLC compared to ILC patients [29].

#### 2.2.2. Tubulolobular Carcinoma

Tubulolobular carcinoma is histologically similar to the classic subtype but has a portion of tubules among the lines of malignant cells. The expression of E-cadherin by the components of tubulolobular carcinoma suggests that tubulolobular carcinoma should be regarded as ductal rather than lobular carcinoma [30,31,32]. ILC with tubular elements exhibits non-cohesive neoplastic cells (due to the absence of E-cadherin and β-catenin) among those with cohesive tubular elements (due to the presence of β-catenin that retains the Adherens Junctions) [20]. 

#### 2.2.3. Alveolar and Solid-Papillary ILC

This subtype displays an accumulation of >20 malignant cells in a glomerular-like formation surrounded by thin layers of collagen fibers and vessels reminiscent of alveoli [33]. E-cadherin may be expressed and loss of α-, β- and γ-catenins, as well as re-localization of p120-catenin, may lead to altered cell adhesion [34]. At this subtype, giant osteoclastic cells may be present. The solid variant of ILC resembles mammary primary non-Hodgkin lymphoma, making differential diagnosis between those diseases difficult. It manifests as sheets of cells morphologically similar to the classic ILC cells, with little stromatic tissue intervening in the described structure. It has also been referred to as confluent ILC. Prognosis may be poorer than classic ILC [19]. On the other hand, solid-papillary ILC is described as a well-circulated tumor with fibrous pseudocapsules and classic satellite ILC foci accompanying it (both types of tumors have lost E-cadherin) [35]. 

#### 2.2.4. Trabecular and Plexiform ILC

This one is an ambiguous entity; many pathologists include this form within classic ILC. The thought behind the presentation of this form as an individual entity is its further classification depending on the one-, two- or three-cell-thick layering of the malignant cells that follow the cytomorphology of the classic ILC’s cells [33]. Plexiform carcinoma is another dubious term used to refer to a coarse formation of tumor cells in clusters with loose junctions resembling a net [20]. 

#### 2.2.5. Histiocytoid and Neuroendocrine ILC

Histiocytoid ILC displays some individually existing and some single-cell layers. These observed tumor cells appear to have nuclei without significant atypia and abundant cytoplasm with a ground-glass appearance. This subtype usually features ER/PR−, lack of E-cadherin expression, expression of AR and GCDFP15, overexpression of HER2, apocrine change, + immunohistochemistry for cytokeratins (CK7, CK8/18) and histiocytic cell markers (PGM1) [36,37,38,39]. It can be confused with benign cell neoplasia. ILC with extracellular mucin is described as a configuration consisting of 80% classic ILC and 20% signet ring cell carcinoma lying on abundant extracellular mucin (both tumors lack E-cadherin). ILC with neuroendocrine features represents the subtype of ILC that expresses one or more neuroendocrine markers and proliferates in a way of making nests; the chromatin in the nuclei feature density variation (salt and pepper appearance) [20].

### 2.3. Associated Mutations and Mechanisms of Disease Progression

**Figure 2:**
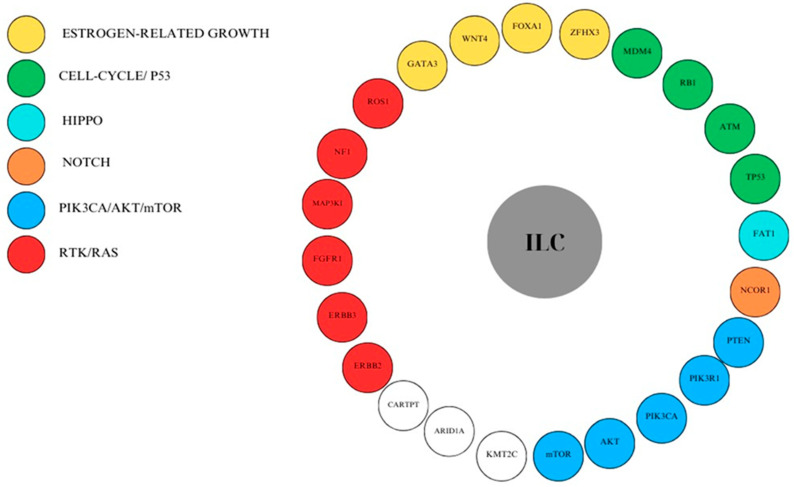
Genetic mutations linked to ILC, and signaling protein pathways involved.

The most important genetic mutations associated with ILC tumors are described below. Table 1 provides a summary of these mutations and their outcomes. The mutated genes are categorized according to their function in Figure 2.

#### 2.3.1. *CDH1* Mutations

Importantly, a variety of gene mutations drive the formation and progression of ILC and influence the biological features of the tumor, the likelihood of cure, and the possibility of relapse. Mutations of CDH1 are the most frequently detected mutations in ILC and are characteristic of this subtype of breast cancer. E-cadherin is disrupted, reducing cell–cell adhesion and leading to ILC’s signature pattern of single-file invasion through epithelial tissues [6]. The distinct pattern of spread of ILC, through involvement of the gastrointestinal tract, peritoneum and ovaries, is associated with *CDH1* mutations. While *CDH1* loss is characteristic of ILC and correlates with an initial low tumor grade, the mutation also serves as a significant long-term contributor to metastasis and poor long-term survival rates despite often being diagnosed in early stages of disease [47].

Mutations that result in the loss of E-cadherin (mostly *CDH1* mutations) and cytoplasmic localization of p120 catenin are specific for ILC and can be a useful diagnostic tool to differentiate between ILC and NST [13]. Christgen et al. reported E-cadherin loss in 95% (40 out of 42) of ILCs and only 1% of invasive NST carcinomas. *CDH1* mutations were significantly more prevalent in ILC, occurring in 76% of cases, while only 1% of NST displayed *CDH1* mutations. The majority of *CDH1* mutations were of the frameshift or nonsense type, causing the generation of premature stop codons. When *CDH1* mutations were present, 91% of breast carcinomas lacked E-cadherin expression, and the remaining 9% with *CDH1* mutations maintained E-cadherin expression, all involving missense mutations [48]. While germline *CDH1* mutations are infrequent in individuals with ILC, occurring in less than 1% of cases, they seem to be more prevalent (8%) in women who have bilateral ILC. According to the Consortium of Investigators of Modifiers of BRCA1/2 (CIMBA), only 2.2% and 8.4% of patients with *BRCA1* and *BRCA2,* respectively, revealed ILCs [49]. Nonetheless, the presence of germline mutations in *BRCA2* and *CDH1* is linked to a significantly elevated risk of ILC. On the contrary, germline mutations in *ATM*, *CHEK2* and *PALB2* are associated with a moderately increased risk of developing ILC [16]. *BRCA2* and other DNA damage response (DDR) gene mutations (*ATM*, *CHEK2*, *PALB2*) are present in a small subset of ILC cases and are associated with increased genomic instability [50]. Such mutations are associated with a higher risk of metastatic disease and are less favorable with regard to survival. *BRCA2* and other DDR mutations drive aggressive disease and metastasis, leading to an adverse prognosis for patients whose tumors carry these alterations. PARP inhibitors and platinum-based therapies may be effective for DDR-mutant ILC [50].

More specifically, the *CHEK2* gene, involved in DDR, has been implicated in a subset of breast cancers, including ILC. Mutations in this gene can compromise the DNA repair process and are associated with increased breast cancer risk. *CHEK2* mutations may correlate with a more aggressive disease course, although data specifically for ILC are limited. This mutation might also indicate a potential response to therapies that exploit DNA repair deficiencies [51,52,53].

Sijnesael et al. found that the loss of E-cadherin led to the translocation of p120, causing instability in classical cadherin family members, disrupting RhoA-dependent actomyosin contraction during cell division (cytokinesis) and affecting gene regulation through Kaiso (ZBTB33), a protein with gene regulation functions [51]. Kaiso can suppress mRNA transcription by binding to the canonical Kaiso-binding sequence (cKBS), TCCTGCNA, or it can have noncanonical functions by interacting with the CGCG-containing consensus KBS, TCTCGCGAGA [37,52]. The nuclear translocation of p120 played a role in alleviating Kaiso-dependent transcriptional repression. Sijnesael’s team explored a specific transcriptional program in ILC tumors that were resistant to anoikis (cell detachment-induced apoptosis) and termed it the Anoikis Resistance Transcriptome (ART). They identified a 33-gene signature, the Kaiso-specific ART (KART), which was positively associated with histological ILC breast cancer. High KART signatures were linked to a favorable prognosis in breast cancer, regardless of the histological type. Additionally, a gain of function mutation of HER 2 (*ERBB2* mutation) resulting in HER 2 amplification—although uncommon in ILC—was associated with higher-grade ILC tumors and early relapse [13,54]. 

#### 2.3.2. *FOXA1*

*FOXA1* (also called HNF3α), a crucial ER transcriptional modulator, enhances chromatin accessibility for ER by coordinating DNA binding within a protein complex [55,56]. In the study of Ciriello et al., *FOXA1* mutations were observed in 7% of ILC cases (9/127), clustering within the fork-head DNA binding domain (FK), particularly in the W2 loop, creating a mutation hotspot (MSH) unique to ILC and less common in IDC [47]. These mutations appear to maintain *FOXA1*’s DNA-binding ability, as only a few DNA-interacting residues were altered. Increased *FOXA1* mRNA levels and decreased DNA methylation at *FOXA1* binding sites in mutant cases support a functional correlation with transcriptional activity [47].

ILC also showed enrichment for *FOXA1* mutations but fewer *GATA3* mutations in another ER modulator, GATA3, than IDC. ILC had significantly lower GATA3 mRNA and protein levels than IDC (*p* = 0.007 and *p* = 2 × 10^−4^, respectively), suggesting a unique reliance on FOXA1 rather than GATA3 for ER modulation in ILC [47,57]. Thus, these mutation patterns underscore distinct ER regulatory mechanisms in ILC versus IDC.

#### 2.3.3. PTEN and PIK3CA

In ILC tumors, mutations in the phosphatase and tensin homolog (*PTEN*) and *PIK3CA* genes are common. PIK3CA mutations are present in ~30–50% of ILC cases, promoting cell growth and survival through the PI3K/AKT pathway. Mutations in PIK3CA are associated with more indolent tumors but also contribute to endocrine resistance. These mutations are generally associated with a more favorable tumor grade but can complicate treatment due to endocrine resistance. Patients with PIK3CA mutations may benefit from PI3K inhibitors (e.g., alpelisib), especially when combined with endocrine therapy. However, response rates are variable, and PIK3CA status alone may not predict treatment efficacy [58,59]. Conversely, mutations in *PIK3R1*, *AKT1*, *AKT2* and *mTOR* are less frequent in ILC. Interestingly, it has been observed that *PIK3CA* mutations (occurring in 48% of cases) and *PTEN* mutations (occurring in 13% of cases) tend to be mutually exclusive in ILC, whereas mutations in *GATA3* are more prevalent in NST [60]. *ERBB3* alteration frequency has been investigated in many studies, although only Zhu et al. found *ERBB3* somatic mutation in primary pleomorphic ILC (4/17 patients) [42]. The higher rate of HER2 expression in pleomorphic ILC was linked to a poorer prognosis compared to classic HER2− ILC. On the contrary, *ERBB2* alteration frequency (mutations and amplifications) was clarified by a large number of studies [44,61,62,63,64,65,66,67].

Ribnikar et al. concluded that approximately 50% of ILC tumors have been found to exhibit alterations in the PIK3CA-PTEN-AKT1 signaling pathway [46]. This research has linked these mutations to characteristics like lower histological grade and a lower Ki-67 index, which measures cell proliferation [46]. Notably, *PIK3CA* gene mutations are the most common somatic genetic changes in ER+ BC and are associated with favorable features, such as smaller tumor size, lower grade, estrogen receptor positivity and older patient age [46]. When PIK3CA-activating mutations are present in primary ILC, this is linked to longer periods without distant metastasis and overall survival. Lastly, there is a greater benefit observed with aromatase inhibitors (AIs) and extended endocrine therapy compared to the standard 5-year treatment with tamoxifen [46]. Furthermore, studies have shown that the co-occurrence of *AKT1* mutations with alterations in other genes (like *PIK3CA*) can significantly impact treatment decisions and prognostic outcomes. Conversely, PIK3R1, AKT1, AKT2 and *mTOR* mutations are less frequent in ILC. Interestingly, it has been observed that *PIK3CA* mutations (occurring in 48% of cases) and *PTEN* mutations (occurring in 13% of cases) tend to be mutually exclusive in ILC, whereas mutations in *GATA3* are more prevalent in NST [60].

*ERBB3* alteration frequency has been investigated in many studies, although only Zhu et al. found *ERBB3* somatic mutation at primary pleomorphic ILC (4/17 patients) [42]. The higher rate of HER2 expression at pleomorphic ILC was linked to a poorer prognosis compared to the classic HER2− ILC. Unlike HER2-amplified tumors in invasive ductal carcinoma (IDC), ILC often harbors *ERBB2* mutations instead of amplification. *ERBB2* mutations drive growth independently and are associated with APOBEC mutagenesis, which promotes endocrine resistance in ILC. Patients with *ERBB2* mutations often face challenges with endocrine therapy, leading to poorer survival due to resistance. HER2-targeted therapies, such as neratinib, can benefit ILC patients with these mutations [58,68]. Unlike ERBB2 alteration frequency, both mutations and amplifications have been clarified by many studies [44,61,62,63,64,65].

Ciriello et al. revealed that ILC exhibits the highest average levels of Akt activation, as measured by phospho-Akt and PI3K/Akt signaling, across all breast cancer subtypes. This makes targeted inhibition of the PI3K/Akt pathway a promising therapeutic strategy for ILC [47].

#### 2.3.4. FGFR1

*FGFR1* is the primary genetic amplification in the 8p12-p11.2 region for individuals with ILC, and it has been demonstrated that blocking FGFR1 effectively hinders cell growth in preclinical models [40]. When *FGFR1* is overexpressed in breast cancer cell lines, it leads to resistance to tamoxifen [69]. Conversely, when *FGFR1* is silent, it heightens the sensitivity to tamoxifen. Furthermore, mutations and amplifications in *FGFR* have been linked to the recurrence of the disease, and irregularities in the *FGFR* family of proteins are implicated in the poor outcomes observed in patients with ILC [13]. Amplifications in *FGFR1* and *FGFR2* are involved in endocrine resistance and provide an alternative signaling route for tumor proliferation. This pathway is upregulated in approximately 10% of ILC cases. *FGFR* amplifications contribute to an aggressive disease profile and poorer prognosis in endocrine-resistant ILC. FGFR inhibitors are under investigation for potential benefits in treating *FGFR*-amplified, endocrine-resistant ILC [70,71]. Additionally, there are single-nucleotide polymorphisms (SNPs) associated with a low predisposition to breast cancer, and these SNPs have varying impacts on the occurrence of ILC compared to invasive NST tumors. Specifically, a specific SNP located at 7q34 has been identified as influencing the development of ILC [16].

Moreover, the Int7G24A variant of Transforming Growth Factor-β Receptor Type I has been linked to the occurrence of invasive BC [72]. Mutations in *TGFBR2* are noted in a subset of ILC cases and are involved in the TGF-beta signaling pathway, which regulates processes such as cell growth, differentiation and apoptosis. Alterations in this pathway can lead to increased tumorigenicity and invasiveness. *TGFBR2* mutations may correlate with aggressive tumor behavior and poorer clinical outcomes, suggesting that patients with such alterations might require closer monitoring and aggressive treatment strategies [72]. Targeting TGF-beta signaling may offer therapeutic avenues for ILC patients with *TGFBR2* mutations, although specific therapies are still experimental. In their study, Chen et al. found no somatic mutations in the TGFBR1 gene related to breast cancer. However, they did establish a strong correlation between an inherited variant in TGFBR1 and patients facing advanced stages of breast cancer [72].

#### 2.3.5. APOBEC-Derived Mutations

Apolipoprotein B mRNA-editing catalytic polypeptide (APOBEC)-induced mutations are caused due to base-pair substitutions (C to G transitions and C to G transversions) most commonly in TCA or TCT triplets, thus changing codons in the transcriptional DNA clone. This mechanism leads either to amino acid changes, or deactivation of tumor suppressive and ER-related genes, due to stop codon induction [14]. All of the above contribute to tumor growth and endocrine resistance in ER+HER2- BC. In the setting of early BC, APOBEC-driven inactivating mutations are reported in *CDH1*, *NCOR1*, *TP53* and *MAP3K1* genes. Specifically, for ILC, APOBEC-induced *CDH1* mutations were the only deactivating mutation in 6% of the tumors [14]. In the metastatic setting, although less frequent, these mutations remained. However, mutations in genes such as *BRCA2*, *ARID1A* and *KMT2C* were reported only in metastatic tumors, implying evidence of their contribution to disease progression. Apart from *ARID1A* and *KMT2C*, *ZFHX3* and *NF1* are also associated with endocrine resistance in metastatic disease. Lastly, helical *PIK3CA* mutations are more frequent in metastatic compared to primary tumors, providing an advantage to the first by over-activating PIK3 signaling [14]. 

Moreover, ILC APOBEC-induced TP53 mutations, found in 5–15% of ILC cases, are less common in classic ILC but more prevalent in pleomorphic ILC, a more aggressive variant. This mutation is associated with higher tumor grade, increased genomic instability and poorer prognosis. Tumors with TP53 mutations have a more aggressive phenotype, contributing to lower survival rates and resistance to some treatments. TP53 mutations may indicate the need for more intensive treatment approaches due to associated chemoresistance [47,73].

Also, ILC APOBEC-induced *MAP3K1* mutations occur in about 15% of ILC cases. This gene is involved in MAPK signaling pathways, which play critical roles in cellular responses to growth factors and stress. *MAP3K1* mutations can lead to alterations in tumor cell proliferation and survival [47]. While the exact impact of *MAP3K1* mutations on survival in ILC remains less clearly defined, their presence is often correlated with specific tumor characteristics, including a lower grade and potentially better responses to endocrine therapy. Understanding the specific alterations in *MAP3K1* could help tailor treatments that modulate MAPK pathway activity in affected patients [47].

#### 2.3.6. ESR1, MDM4

*ESR1* gene mutations are mainly found in metastases from ER+ and HER2− ILC tumors, suggesting that they may be acquired mutations [74]. *ESR1* mutations in ILC usually arise in response to prolonged endocrine therapy. These mutations lead to constitutive estrogen receptor activation and endocrine resistance, often in advanced or metastatic disease. They are associated with a more aggressive disease course and poorer survival, especially in endocrine-resistant cases. Combination therapies, such as CDK4/6 inhibitors with anti-estrogens, are commonly used to manage *ESR1*-mutant ILC [75,76]. However, there is a lack of comprehensive studies comparing matched primary and metastatic samples for eliminating the possibility that *ESR1* mutations identified in metastatic disease were already present in a rare subclone of the primary tumor. Fumagalli et al. examined matched primary and metastatic ER+ samples from 37 patients and noted that none of the four *ESR1* mutations identified in the metastatic samples was detected in the corresponding primary tumor samples [77]. Cao et al. confirmed the theory of Desmedt et al. about the frequent *ESR1* copy number gain and amplifications and associated it also with higher recurrence risk [44,74]. This genetic profile may inform treatment strategies with tyrosine kinase inhibitors (TKIs) and trastuzumab [78]. Finally, Cao et al. reported the amplification of *MDM4* (*MDMX*), primarily known as a negative regulator of *TP53* in ILC [44].

#### 2.3.7. Endocrine Resistance-Associated Mutations

Specific mutations are correlated with a low response to ET. This is defined as a relapse within 2 years of adjuvant endocrine treatment or disease progression during the first 6 months of first-line endocrine therapy. These mutations lead to reduced expression of ERα and increased expression of estrogen-related receptor γ (ERRγ). Therefore, changes in gene expression activate AP1-dependent transcription, upregulate the mitogen-activated protein kinase (MAPK)/extracellular signal-regulated kinase (ERK) pathway and activate the glutamate receptor, leading to reduced tamoxifen sensitivity [79]. High sterol regulatory binding element protein-1 (SREBP-1) expression, which boosts the function of fatty acid synthase, leads to an insufficient response to the aromatase inhibitor letrozole [80]. Moreover, WNT4, a ligand in the WNT signaling pathway, seems to drive estrogen-induced growth and can be involved in endocrine resistance in ILC [81]. Additionally, *FOXA1* (ER transcription modulator) is also correlated with cancer progression and the development of endocrine resistance, due to its co-expression with ER at high levels in endocrine-resistant metastatic breast cancer [13,81,82,83].

#### 2.3.8. Prognostic Value of Genetic Alterations

ILC is also characterized by increased gains in chromosomes 1q, 8q and 16p along with losses of 8p23-p21, 11q14.1-q25 and 16q and amplifications of 1q32, 8p12 and 11q13 compared with NST [60]. As a result, utilizing molecular assays specifically designed for ILC may enhance prognostic accuracy compared to currently available gene expression assays—Prosigna Breast Cancer Prognostic Gene Signature Assay (PAM50), intrinsic subtyping and risk of recurrence (ROR) score, Oncotype DX breast recurrence score (RS), MammaPrint and EPclin risk stratification for emerging distant metastasis, Breast Cancer Index (BCI) recurrence prediction and GenomicG (GG) for a better reclassification of breast tumors [60]. A recently introduced 194-gene signature, known as LobSig, has demonstrated effectiveness in predicting prognosis for ILC. LobSig stands out as the first gene signature specifically designed for the prognostic assessment of ILC patients and exhibited superior performance compared to the above stepwise, multivariate Cox proportional hazards model, particularly in Grade 2 tumors [21,60]. Tumors with high LobSig expression were notably linked to a higher prevalence of mutations in *ERBB2* (20.0%), *ERBB3* (14.3%), *AKT1* (8.6%), and *ROS1* (8.6%), suggesting potential avenues for targeted therapeutic interventions and answering the question whether chemotherapy is beneficial for ILC patients; however, the study was not strong enough to provide reliable results. In addition, LobSig needs to undergo further validation in ILC patient cohorts [60].

#### 2.3.9. ILC mRNA Subtypes and Prognosis

Using mRNA-seq data from LumA ILC samples (*n* = 106), Ciriello et al. identified three ILC subtypes: reactive-like, immune-related, and proliferative [47]. A 60-gene classifier scored all ILC samples in TCGA (*n* = 127) and METABRIC datasets, revealing key genomic features distinguishing each subtype, though without specific somatic mutations or DNA copy-number changes. Reactive-like tumors showed a high expression of epithelial and stromal-associated genes (e.g., *EGFR, MET*), immune-related tumors had elevated immunogenic signaling genes (e.g., IL, TCR), and proliferative tumors had a low expression of these genes but higher proliferation rates [47]. Proliferative tumors also expressed cell cycle and DNA repair proteins, while reactive-like tumors had higher c-Kit and PKC levels, and immune-related tumors showed elevated immune modulators like STAT5 [47].

Further analysis indicated reactive-like ILC patients had significantly better disease-specific (DSS) and overall survival (OS) than proliferative ILC patients in the METABRIC dataset. These findings suggest the reactive stromal phenotype aligns with favorable prognosis, while higher proliferation indicates a worse outcome in luminal/ER+ breast cancers, which is consistent with prior research [84].

### 2.4. Advances in Treatment and Prospects

#### 2.4.1. Surgical Considerations

Truin et al. specifically reported that the histological type of tumor is an independent factor for the use of BCS versus mastectomy, as 82.2% of ILC patients were scheduled for mastectomy, in contrast to 62.5% of IDC patients (*p* < 0.0001). Moreover, reoperation was more frequent among ILC patients (8.2%) due to re-excision of surgical margins, compared to IDC (3.4%) (*p* < 0.0001) [85]. Last but not least, salvage surgery may be more frequently considered for ILC rather than IDC patients (*p* < 0.0068) [86].

#### 2.4.2. Adjuvant Radiation Treatment

Adjuvant whole-breast radiation therapy after clear-margin BCS has reported adequate local control both for ILC and non-specific type breast carcinomas [16]. Due to the multifocal nature of the disease, accelerated partial breast irradiation brachytherapy is associated with higher rates of ipsilateral breast tumor recurrence compared to IDC (*p* < 0.008). It is therefore not recommended [87].

#### 2.4.3. Chemotherapy

**Figure 3:**
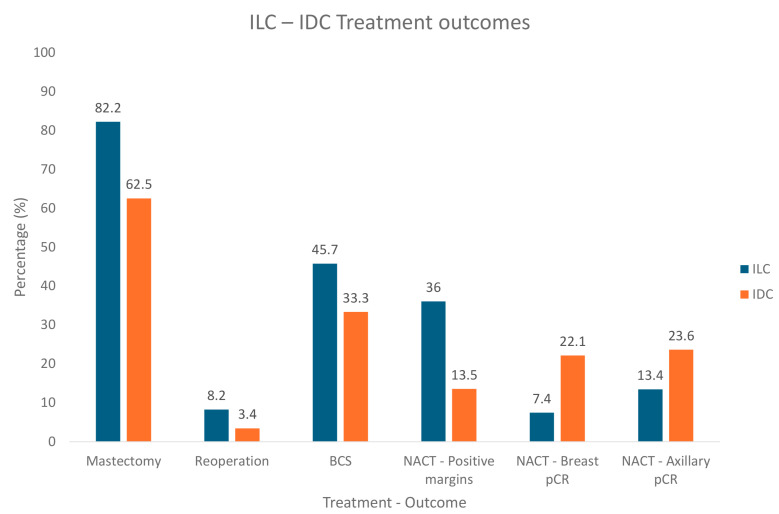
Comparison of treatment strategies and therapeutic outcomes between ILC and IDC patients. Abbreviations: ILC: Invasive Lobular Carcinoma; IDC: Invasive Ductal Carcinoma; BCS: breast conservation surgery; NACT: neo-adjuvant chemotherapy; pCR: pathologic complete response.

Despite the histological and molecular differences between ILC and NST, current guidelines take no consideration of the histological subtype in the prescription of neo-adjuvant chemotherapy (NACT) in BC. However, a recent meta-analysis suggests that, compared to NST, ILC patients are less likely to achieve breast and axillary pathologic complete response (pCR) (22.1% vs. 7.4% and 23.6% vs. 13.4%, respectively; *p* < 0.0001), to undergo breast conservation surgery (BCS) (45.7% vs. 33.3%, respectively; *p* < 0.0001) and more likely to have positive margins postoperatively (36% vs. 13.5%, respectively; *p* < 0.0001), after NACT [88]. A brief comparison of treatment outcomes between ILC and IDC is provided in Figure 3. More research is essential to fully understand the benefit of NACT in ILC patients, to provide tailored treatment. Considering neo-adjuvant chemotherapy regiments, the GAIN III trial compared intense dose-dense chemotherapy to tailored dose-dense chemotherapy for high-risk early breast cancer patients. The intense dose-dense regimen included epirubicin, nab-paclitaxel and cyclophosphamide (three cycles of each), whereas the tailored strategy included four courses of epirubicin and cyclophosphamide, followed by four courses of docetaxel. Although both strategies had similar invasive disease-free survival (iDFS) at 4 years, luminal-B and lobular histology reported superior iDFS rates with the tailored approach. Additionally, the tailored NACT group presented fewer adverse events (especially hematological), compared to the intense approach, as an escalation of therapy was based on toxicity and complications [89]. It could therefore be a feasible therapeutic plan for luminal-B and ILC patients. 

According to a pooled analysis of the PLAN B and SUCCESS C trials, anthracycline regimens provide no additional benefit and more adverse events when it comes to HER2−, high-risk breast cancer, compared to docetaxel/cyclophosphamide regimens. However, pN2/N3 ILC patients report better DFS rates with anthracycline-containing regimens, which are therefore recommended for these patients [90]. The benefit of adjuvant chemotherapy (ACT) is also a controversial topic. A recent meta-analysis by Trapani et al. provided evidence that ACT, in addition to standardized treatment, does not correlate with higher overall survival (OS), compared to no additional ACT in early ILC patients [91]. Interestingly, only one study reported an increase in OS after ACT [92]. 

Lastly, in the metastatic setting, the pooled analysis results of two phase-III studies (305 EMBRACE, 301) and a phase-II study (study 211) suggest that patients with metastatic BC respond equally to eribulin regardless of tumor histology. Specifically, the median OS for ILC and NST patients treated with eribulin was 13.4 and 13.5 months, respectively (*p* = 0.431), and PFS was 4.1 and 3.6 months, respectively (*p* = 0.393). For patients with ER+ and HER2− disease, OS and PFS were also similar [93].

#### 2.4.4. Endocrine Resistance and HER2 Mutations

In the metastatic setting, endocrine treatment (ET) combined with CDK4/6 inhibitors (CDK4/6i) has proven beneficial both for NST and ILC patients, as a pooled analysis of three clinical trials (MONARCH-2, PALOMA-3, MONALEESA-3) suggests an increase in OS after CDK4/6i and fulvestand [94]. The population-based study of Mouabbi et al. explored the outcomes of ET combination treatment based on histology in metastatic BC patients. Patients included in the study had ER+, HER2−, NST or ILC or mixed (ductal and lobular) histology [95]. Combination treatment involved ET plus CDK4/6i (palbociclib), ET plus mTOR inhibitors (everolimus) or ET combined with P3KI inhibitor (alpelisib). The mean PFSs for patients who received AI plus CDK4/6i as a first-line treatment were 16, 18.8 and 16.9 for ILC, NST and mixed, respectively (*p* = 0.675 and *p* = 0.442) [95]. Similarly, mean OS presented no statistically significant differences (38.3, 35.9 and 34.6 months, respectively; *p* = 0.157, *p* = 0.769). Moreover, there was no statistical difference in mean PFS (5.2 and 2.9 months, respectively; *p* = 0.638) and OS (13.7 and 16.4 months, respectively; *p* = 0.674), between NST and ILC patients who received ET plus alpelisb. NST and ILC patients benefited equally from the combination of ET plus everolimus [95]. However, the 5 year OS for patients treated with CDK4/6i and AI was 2.5 fold higher in NST compared to ILC patients (38% and 14%, respectively). This could be due to a higher rate of mutations that result in CDK4/6i resistance in ILC patients. The most important of those are discussed in the next section. Finally, the use of alpelisib is further supported by the SOLAR-1 as a combination to ET. Given that P3KI-AKT are the second most common mutation after CDK1 in ILC tumors, histology-specific trials should be conducted to generalize the benefit of alpelisb in ILC patients [16,95].

However, the eventual development of endocrine resistance mitigates therapeutic options. Dysregulation of APOBEC, which promotes tumor mutagenesis and heterogenicity, is more frequent in ILC and metastatic tumors and promotes resistance to ET. Therefore, the efficacy of fulvestrant combined with CDK4/6i is inferior in APOBEC+ tumors, compared to APOBEC− tumors, as APOBEC+ was associated with shorter time-to-treatment discontinuation compared to APOBEC− (7.8 vs. 12.1 months, respectively; *p* = 0.036) [96]. Seeking alternatives to ET and following the hypothesis that a group of ILC tumors express immune-related genes and immune checkpoints, the GELATO trial examined the benefit of PD-L1 blockade, following carboplatin as immune induction. Reportedly, a clinical benefit of 26% was observed with a median OS of 54.4 weeks. This is due to 6 of 23 patients presenting partial response or stable disease. Interestingly, four of six patients presented triple-negative ILC [5]. Another alternative could be the addition of riluzole, which induces apoptosis and ferroptosis in tamoxifen-resistant ILC tumors in vitro and ex vivo, in patient-derived extracts with the combination of fulvestrant. These promising results necessitate larger laboratory studies and potentially clinical trials to assess the therapeutic potential of riluzole [97]. Another alternative to ET-resistant ER+, HER2− metastatic BC is using antibody–drug conjugates (ADCs). The TROPiCS-02 trial is a phase III clinical trial assessing the efficacy of Sacituzumab govitecan (SG), an ADC comprising an antibody against Trop-2 bound to SN-38, a metabolite of irinotecan [95,96]. Trop-2 is a transmembrane calcium signal transducer, common in metastatic BC. The study concludes that SG offers an increase in OS compared to chemotherapy (14.4 vs. 11.2 months, respectively; *p* = 0.020), an improvement in ORR (21% vs. 14%, respectively; *p* = 0.035) [98,99]. Regarding triple-negative metastatic BC, the study of Bardia et al. compared PFS, OS and tolerability between SG and chemotherapy without regard to tumor histology. Treatment with SG increased PFS (5.6 vs. 1.7 months; *p* < 0.001) and OS (12.1 vs. 6.7 months; *p* < 0.001, respectively) compared to chemotherapy. The most common treatment-related adverse event for SG and chemotherapy was neutropenia (63% and 43%, respectively) followed by anemia (34% and 24%, respectively) [100]. Clinical trials specific to metastatic ILC could explore the benefits of SG in this BC subtype.

*HER2*-mutated, non-amplified breast cancer (HER2mut) is also a distinct mechanism of ET resistance. Combined with the fact that they occur predominantly on ER (+) tumors and that neratinib alone performed poorly on *HER2mut* tumors, the MutHER study compared the efficacy of neratinib plus fulvestrant versus neratinib alone, in *HER2mut*, non-amplified metastatic breast cancer. The neratinib–fulvestrant cohort presented clinical benefit rates (CBRs) of 38% median PFS of 24 weeks and was not correlated with prior fulvestrant treatment. ILC patients reported significantly higher CBRs (61.5%, *p* < 0.02), and *TP53*, *PIK3CA*, *CDH1*, *ESR1* and *RB1* mutations were identified but not associated with CBR. *HER* L755 mutations (L755S and L755_ins del) were correlated with lower CBRs, compared to other *HER2* mutations, contrary to an exon 20 insertion, which presented a higher CBR (83.3%, *p* = 0.01). Interestingly, a D769Y mutation presented variations during treatment, as it was undetectable in cycle 2 and redetected in cycle 9, with its levels increasing at progression [15]. In conclusion, the efficacy of neratinib plus fulvestrant and trastuzumab is further supported by the SUMMIT trial, making this combination the standard for HER2mut metastatic breast cancer (MBC), with prior CDK4/6i therapy [101]. Also, *ERBB2* mutations were detected 3.5-fold more frequently in ILC tumors with sustained cell proliferation (Ki-67 > 10%) after short-term neoadjuvant ET (NAET) [45]. Conversely, failure to achieve adequate suppression of cell proliferation was correlated with *ERBB2* mutated, non-amplified breast cancer, in ILC [72]. 

#### 2.4.5. HER3-Targeting Therapies and Prospects

Mishra et al. show that *HER3* mutations in the ECD provide resistance to lapatinib in vitro. Although neratinib at a concentration of 50 nM did not reverse this effect, increasing the concentration tenfold suppressed colony formation in the cell line MCF10AHER2. Therefore, a higher dose of neratinib could surpass resistance in *ERBB3* mutated tumors. However, this regimen would be poorly tolerated by patients due to side effects such as diarrhea [102]. A clinical trial assessing the use of neratinib in combination with everolimus, palbociclib or trametinib (NCT03065387) in solid tumor patients with *EGFR* or *HER2* mutation/amplification, and *HER3/4* and *KRAS* mutation is currently active. Based on the results of this study, crucial insight can be gained about the tolerability of neratinib combination treatment and the role of the underlying mutations on treatment resistance. BC- and ILC-specific studies could also be conducted to generalize the results in *HER2*- and *HER3*-mutated tumors [102].

Lastly, anti-HER2 therapy may be effective in metastatic breast cancer with somatic *HER3* mutations, as a case report by Bidard et al. suggests. This concerns an ER+, HER2−, HER3 G284R, NST tumor with liver metastasis, treated with lapatinib and trastuzumab. The HER3 G284R and *TP53 R282W* mutations were common for primary and metastatic tumors [103]. The double HER2 blockade resulted in a complete metabolic response in 2 weeks and the patient remained disease-free 40 weeks after surgical resection of the liver metastasis. Although this is a case report, these promising results can be generalized through clinical trials specific to advanced ER+, HER2−, and HER3 mutated tumors [103].

#### 2.4.6. Resistance to CDK4/6 Inhibitors

Although CDK4/6is are commonly used as a first-line treatment for ER+, HER2− BC, the disease usually progresses due to resistance acquisition. The most important resistance mechanisms described in the literature are the absence of retinoblastoma protein due to *RB1* loss, cyclin E1, E2 and CDK2 upregulation, PI3K/AKT/mTOR pathway upregulation, *TP53* mutations, CDK6 upregulation and loss of *FAT1* function. These mechanisms have been associated with resistance to palbociclib and ribociclib and are targetable alterations to distinguish CDK4/6 sensitivity. As overlap with endocrine resistance is common, alternatives such as mTOR inhibition and apoptosis induction should be sought after [17,41,43,104].

#### 2.4.7. Tyrosine Kinase Inhibitors

Apart from neratinib, other tyrosine kinase inhibitors (TKIs) report therapeutic potential in *HER2mut* ILC. Poziotinib suppresses the proliferation of *HER2mut*, non-amplified L755S cells at lower dosages compared to neratinib (IC50: 0.3 vs. 1.3 nmol/L, respectively); however, they presented similar inhibition of p-Akt protein and neratinib was more effective in inhibiting p-p44/42 MARK signal, in vitro. In vivo, poziotinib reduces the size of primary as well as metastatic tumors (ovaries) in test subjects [105]. Additionally, Tian et al. presented a case of metastatic ILC harboring D769H and *V777L* mutations, resistant to previous chemotherapy and CDK4/6 treatment. Clinical benefit was reported due to the partial response and stability of the disease after the prescription of pyrotinib combined with T-DM1, exemestane and zoledronic acid [106]. 

## 3. Conclusions

In conclusion, the optimal management of ILC tumors necessitates a better understanding of its intricate pathological and genetic landscape. Extensive research concerning gene mutations in ILC has shed light on the mechanisms of disease progression and metastatic spread and the acquisition of drug resistance. Most importantly, the role of *HER2* mutations and APOBEC-derived mutations is crucial in the development of ET resistance. The co-existing CDK4/6i resistance further mitigates treatment options. The identification of drug resistance might therefore have a pivotal role in determining therapeutic plans and providing tailored treatment with optimal outcomes. Moreover, molecular assays utilizing ILC-specific targets, such as gains or losses in chromosomes 1, 8, 11 and 16, along with LobSig expression, can increase prognostic value, compared to current assays. Last but not least, treatment resistance increases the need for alternative strategies and creates new research fields. Large-scale clinical trials should emphasize optimal timing and chemotherapy regimens, the role of PD-L1 blockades and riluzole, and the effectiveness of pyrotinib and poziotinib to maximize our understanding of their potential.

## Figures and Tables

**Table 1:** Summary of ILC genetic mutations associated with disease progression, drug resistance and prognosis.

Study/Publication Year	Population (N)	ER, PR Status	HER2 Expression	Mutations and Frequency	Outcome
Reis-Filho et al. (2006) [40]	13	+/46%+	−	*FGFR1* amplification (8p12-p11.2 region) (46.1%)	MDA-MB-134 cell survival in classic ILC
Malorni et al. (2016) [41]	757	+/+	−	RB1 (32.8%)	CDK4/6i resistance
Zhu et al. (2018) [42]	16	−/−	+	*HER3* (somatic) (24%) *IRS2* (29%)	PILC variant; poor prognosis PILC variant-increased invasion and glucose uptake; poor prognosis; high metastatic potential; reduced DFS, OS
Li et al. (2018) [43]	348	+/data not available	−	*FAT1* loss of function (100%) *TP53* (65%)	CDK4/6i resistance
Cao et al. (2019) [44]	70	+/data not available	−	*ESR1*: CN gain (14%) amplification (10%)	High risk of recurrence
Yadav et al. (2021) [12]	64,609	95%+/80%+	92% −	*CDH1* (germline) (0.77%) *BRCA2* (germline) (3.28%) *CHEK2* (2.35%) *ATM* (1.7%) *PALB2* (0.77%)	High risk for ILC Moderate risk for ILC
Bos et al. (2022) [14]	747	+/data not available	−	APOBEC-driven: *CDH1* (16%) *NOCR1* (10%) *TP53* (10%) *MAP3KI* (11%)	Tumor growth; metastasis; endocrine resistance
Grote et al. (2022) [45]	622	+/87%+	HER2mut enriched	*HER2* (7.8%)	Endocrine resistance
Ma et al. (2022) [15]	381	>87%+/>40%+	−/non-amplified	*HER2* L755 (7.32%)	Endocrine resistance; low CBR
Ribnikar et al. (2023) [46]	365	>90%+/>90%+	>90%−	*PIK3CA* (somatic) (45%)	Favorable prognosis; increased DFS, OS; benefit with AΙs and extended endocrine therapy
